# A Modular Adjustable Transhumeral Prosthetic Socket for Evaluating Myoelectric Control

**DOI:** 10.1109/JTEHM.2020.3006416

**Published:** 2020-07-01

**Authors:** Ben W. Hallworth, James A. Austin, Heather E. Williams, Mayank Rehani, Ahmed W. Shehata, Jacqueline S. Hebert

**Affiliations:** 1 Department of Mechanical EngineeringUniversity of Alberta 3158 Donadeo Innovation Centre for EngineeringEdmontonABT6G 1H9Canada; 2 Division of Physical Medicine and Rehabilitation, Faculty of Medicine and DentistryUniversity of Alberta 3158 EdmontonABT6G 2R3Canada; 3 Glenrose Rehabilitation Hospital 60351 EdmontonABT5G 0B7Canada

**Keywords:** Electromyography (EMG), prosthetics, additive manufacturing, linear discriminant analysis, pattern recognition

## Abstract

Novel myoelectric control strategies may yield more robust, capable prostheses which improve quality of life for those affected by upper-limb loss; however, the development and translation of such strategies from an experimental setting towards daily use by persons with limb loss is a slow and costly process. Since prosthesis functionality is highly dependent on the physical interface between the user’s prosthetic socket and residual limb, assessment of such controllers under realistic (noisy) environmental conditions, integrated into prosthetic sockets, and with participants with amputation is essential for obtaining representative results. Unfortunately, this step is particularly difficult as participant- and control strategy-specific prosthetic sockets must be custom-designed and manufactured. There is thus a need for a system to reduce these burdens and facilitate this crucial phase of the development pipeline. This study aims to address this gap through the design and assessment of an inexpensive and easy-to-use 3D-printed Modular-Adjustable transhumeral Prosthetic Socket (MAPS). This 3D-printed, open-source socket was developed in consultation with prosthetists and compared with a participant-specific suction socket in a single-participant case-study. We conducted mechanical and functional assessments to ensure that the developed socket enabled similar performance compared to participant-specific sockets. Both socket systems yielded similar results in mechanical and functional assessments, as well as in self-reported user feedback. The MAPS system shows promise as a research tool which catalyzes the development and deployment of novel myoelectric control strategies by better-enabling comprehensive assessment involving participants with amputations.

## Graphical abstract

Diagnosing bladder cancer typically involves highly invasive cystoscopy, with followup monitored using uteroscopy. We present a study of the use of dielectrophoresis (DEP) of voided cells from 8 cancer patients and 8 controls as an alternative low-cost and operator-independent method of detection. Results demonstrate that useful DEP spectra can be obtained from the cellular components of a voided urine sample, and that these spectra can detect cancer. The test is of similar efficacy to many more expensive and complex molecular diagnostic methods.

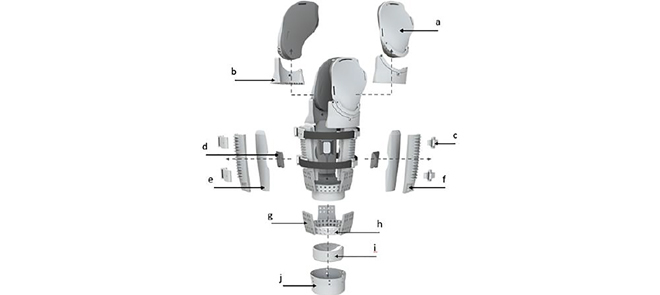

## I. Introduction

Upper limb amputation can pose significant restrictions on everyday activities and overall quality of life. Myoelectric prostheses offer the opportunity to improve upper-limb function and user independence. Recently, upper-limb myoelectric prosthetic research has focused heavily on control and sensory feedback strategies [1]–[3]; however, a crucial link between the user and these systems is commonly overlooked: the prosthetic socket. Wearing a prosthetic socket introduces noise due to factors such as socket fit, sweat, and altered loading of the weight of the terminal device, which must be accounted for in experimentation to obtain results that are most representative of, and thus translatable to clinical use [4], [5]. Therefore, integration of novel control strategies into prosthetic sockets is a crucial stage of experimental evaluation, as the performance of an upper-limb prosthesis is highly dependent upon the consistency of the interface between the user’s socket and residual limb.

A well-designed socket should not only be comfortable for the user, but must facilitate stable prosthetic control by efficiently translating motion of the residual limb to positioning of the terminal device [6]–[8]. A stable socket-residual limb interface is even more crucial for myoelectric prosthesis users, as loss of electrode contact can create electromyography (EMG) artifacts and diminish the quality of user control [9]. Recent innovations in prosthesis design, such as the integration of mechano-, vibro-, and electrotactile feedback systems to provide information on degree of freedom (DOF) control selection [10], grip force [5], [11]–[13], sensation [14], or proprioceptive movement [15] place further reliance on a well-designed socket to create and maintain a stable, direct interface for these systems.

To accommodate a patient’s unique limb morphology and wed the soft tissues of their residual limb with the rigid components of their prosthesis, prosthetic sockets are typically manufactured by a prosthetist on a patient-specific basis [6]. Through this process, a prosthetist aims to achieve a pressure distribution across the residual limb, which achieves the greatest possible comfort, while effectively translating underlying skeletal movement through the stabilization of remaining soft tissues [6]–[8]. Prosthetist-fitted Participant-Specific Sockets (PSS) are typically constructed of a rigid thermoplastic or carbon fibre shell, based on the profile of the residual limb, with or without a prosthetic liner. This rigid, tailored design enables suspension through targeted compression of soft tissue or by creating a suction seal with the wearer’s residual limb. To meet the goals of function and comfort, socket fit must be continually updated to reflect changes in residual limb morphology. This may necessitate manufacturing an entirely new socket [7].

Sockets used in experimental settings often require modifications to fit various input and feedback systems, such as electrodes and tactors, as well as evaluation tools such as pressure sensors. The means by which these systems are integrated depend on the user, the system, and the method of socket suspension and often result in permanent alterations that make the socket unsuitable for use outside of a specific experiment [5]. The cost of a conventional socket can be on the order of a few hundred dollars for duplicate sockets, to thousands of dollars for a completely new fitting. Additionally, there is a variable time-delay associated with fittings [7]; thus, experiments utilizing participants with amputation can require significant time and financial resources, which scale linearly with the number of participants. Even when the time and resources are expended to tailor a PSS system, lingering issues with socket fit have been demonstrated to introduce significant sources of error when quantifying myoelectric control strategy performance. Issues with socket fit may even result in delays or cessation of experimentation due to participant discomfort [16]. Few socket systems exist that facilitate an adaptable geometry, of which yet fewer are specifically designed for upper-limb amputations [17]–[19] (see Appendix A: Existing adjustable socket designs). Only one such system offers the flexibility to accommodate multiple configurations of control or sensory feedback systems, however it is limited to use with able-bodied users with electrodes placed below the elbow [20]. Therefore, there are limited options using existing adjustable socket systems which allow assessments of myoelectric control for participants with proximal upper limb amputation.

These shortcomings motivated the need to develop an experimental analogue to conventional PSS systems. Such a system would serve as a testbed for assessing prosthetic control and function without permanent customization, allowing shared use across multiple participants and experiments over time. To be a viable alternative to a PSS system, this platform must function similarly to a PSS, to minimize confounding variables between different socket systems. The system must also be sufficiently comfortable to be worn for the duration of an experimental session without damaging the residuum. In this work, we address these issues by developing a novel 3D-printed Modular and Adaptable transhumeral Prosthetic Socket (MAPS) and performing a functional comparison against a suction-fit PSS system. We hypothesize that this system will meet the necessary mechanical requirements for laboratory use and facilitate similar user comfort and prosthetic control when directly compared with a PSS system.

## II. Materials and Methods

To define key socket design criteria, critical features of PSS systems were first identified through consultation with clinical prosthetists. From this information, the criteria were divided into subsystems and a series of design proposals for each subsystem were developed. The target parameters of the developed system are summarized in Table 1 [22]. These criteria were fulfilled over the course of the development of the final socket through an iterative design process. The final design was produced after seven whole-system iterations had been developed and assessed on able-bodied participants and on two participants with transhumeral amputation.

**TABLE 1:** Design Requirements for the Modular Adaptable Prosthetic Socket

Criteria Description	Target Specification	Final Design
Length Adjustment	40–100% residual limb length	Achievable with multiple exterior panels
Prosthesis Interface	Compatibility with Bento Arm [21] and mechanical elbow^1^	Criterion met, potential for broader compatibility
Electrode Integration	4 electrodes; flexibility for integration of feedback devices	Up to 7 electrodes; feedback not yet explored
Electrode Placement	4 muscle sites	7 muscle sites
Cost	$500	$125
Fitting Time	<30 minutes	15 min initial fitting; <5min re-don after adjusted to fit
Mass	<1 kg	800g
Static Axial Load Limit	30 N	60 N
Rotational Load Limit	2 $\text{N}\cdot \text{m}$	3 $\text{N}\cdot \text{m}$
Comfort	Comfortable over the course of a research trial (3 hours)	Comfortable for up to 3 hours (user-reported)
Sanitation	Non-porous, cleanable interface surface with limb	All limb-socket interfaces neoprene-lined

^1^ ErgoArm plus, Otto Bock Inc., Germany

### A. Socket Design

**FIGURE 1.**
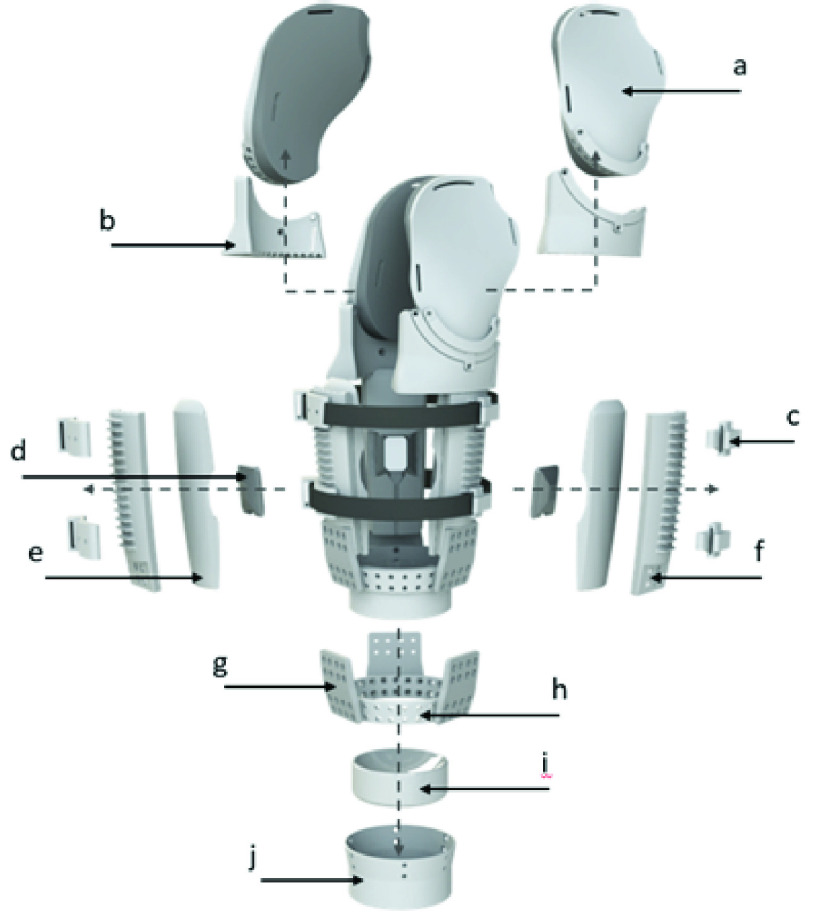
Overview of the Modular Adaptable Prosthetic Socket design and close-up of panel system. a) Exterior shoulder panels & cushion. b) Wing rings. c) Suspension Straps and Mounts. d) Electrode mount. e) Interior cushion panels. f) Exterior panels. g) Flexible Struts. h) Base Ring. i) Distal Support. j) Mounting Plate.

The MAPS was manufactured from primarily 3D printed materials using MakerBot Replicator 2 3D Printers (MakerBot, Inc.) – maximum build size $28.6\times 15.5\times15.3$ cm. All rigid components were constructed from Polylactic Acid (PLA), while neoprene-covered NinjaFlex® thermoplastic polyurethane (Fenner Drives, Inc.) at a 10% infill setting was used to create a compliant, supportive socket-residual limb contact surface. PLA extrusion temperature was 230 °C at 90mm/s, and NinjaFlex® was extruded at 242 °C and 20 mm/s. The socket’s modular design increases compatibility with multiple participants and control/feedback configurations by incorporating components which can be replaced with those of different sizes and in different positions, as indicated below (see also figure 1).

**FIGURE 2.**
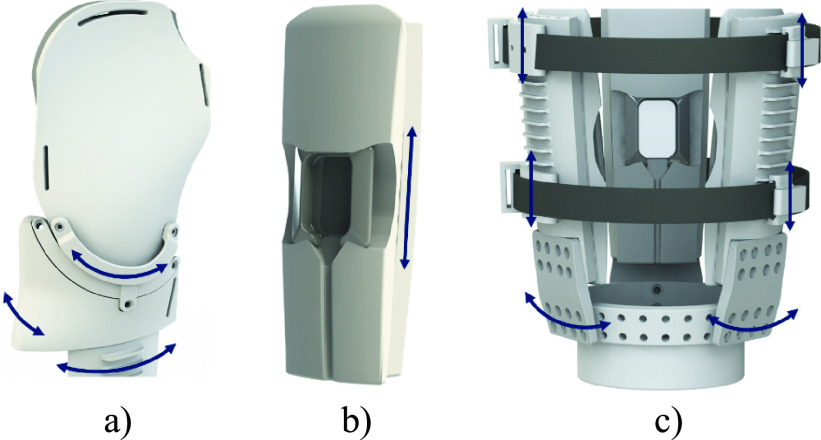
Adjustability of the a) shoulder panel and wing ring, b) interior cushion, and c) socket base.

**FIGURE 3.**
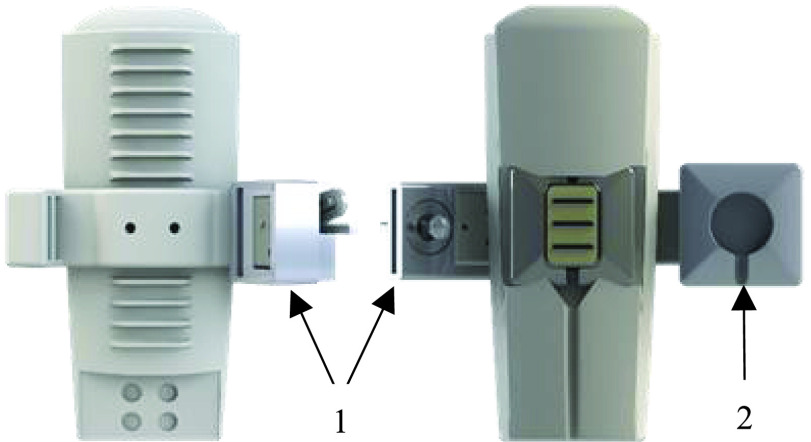
Mechanotactile tactor integrated into circumferential strap assembly (1) and Ottobock Myobock (Ottobock Inc., Germany) electrode integrated into interior panel, with (2) interchangeable mount for Engineering Acoustics C-2 Vibrotactor (Engineering Acoustics, Inc., USA).

a) Shoulder Panels & Cushions. Shoulder panels are lofted inwards to follow the general curvature of the shoulder, allowing the panels to lie against the shoulder and provide suspension against axial translation. The NinjaFlex® cushions lining the entire surface of the wings are convex in shape and compliant to conform to the shoulder and provide proximal point of socket contact under loading, which is stabilized by the rigid exterior shoulder panels. This contact surface resists rotational deviation about the skeletal orientation of the residual limb, with a secondary role to provide axial suspension. Threaded aluminum posts at the top of the exterior panels attach to a shoulder suspension system, securing its axial and transverse rotation.
b) Wing Rings. These shoulder panel mounting components contain alternating holes and concentric curved slots and can be rotated to better conform with the shoulder. Posts on the superior potion of the exterior panels (figure 1e) can be spaced on odd intervals, fixing the rotation of the wings and wing rings, or on even intervals- enabling rotation. One or both panels can therefore be made adjustable as required to conform with shoulder geometry (figure 2a). Shallow slots though the medial face of the wing ring serve as guides for attaching or removing the wing ring as needed while the distal part of the socket remains donned by a participant.
c) Suspension Straps & Mounts. Velcro® (Velcro BVBA) straps wrap the circumference of the socket, to provide compression suspension. A ratcheted buckle system allows the amount of compression to be finely adjusted and maintained. The Velcro® straps allow for various attachments, including electrode and tactor mounts and supporting cushions. Hard plastic mounts anchor these straps to the exterior panels, allowing the position of strap-mounted components to be fine-tuned.
d) Electrode Mounts. These rigid shells slide into cut-out in interior cushion, providing both protection and a stable platform for embedded electrodes. Mounts can be printed with different electrode cut-outs to enable compatibility with various electrode types. One electrode can be housed in each interior cushion (3 total), and one can be mounted to the Velcro strap between each panel (2 spaces * 2 straps) for a total of 7 possible electrodes placed. Additional straps can be mounted to add additional mounting points. These mounting points could also be used to integrate sensory feedback systems, such as mechanotactile tactors [5], [23] and vibrotactile tactors [13], [24] (see Figure 3).
e) Interior Cushions: Cushions provide a compliant fit to conform with and support the wearer’s residual limb. The face contacting the limb is convex in both the radial and axial directions to provide space to avoid any sharp edges of contact along the contact surface. This removes a potential cause of skin irritation. A slot across the cushion surface allows an electrode and mount to be securely embedded, with compression from the interior cushion supported by the rigid exterior panel providing consistent electrode contact against the residual limb (figure 1d). The exterior face of the cushion is designed to fit concentrically within the interior face of the exterior panels, allowing for adjustment of the axial position of each cushion and embedded electrode (figure 2b).
f) Exterior Panels: These panels create a rigid structure for the entire socket, maintain pressure and support across the interior cushions, and provide ridges for mounting suspension straps at various position along the long axis of the socket. The interior face enables attachment of the cushions at various heights using Velcro®. The bottom exterior face has threaded inserts for attaching to a base ring via flexible struts. Panels of different lengths allow residual limbs of varying lengths to be accommodated.
g) Flexible Struts: Flexible struts printed in NinjaFlex® connect the exterior panels to a central base ring. These struts are sufficiently flexible to allow bending inward or outward for doffing and donning, but are rigid to torsion, buckling, and bending tangent to the residual limb, in order to provide stability for the socket panel orientation and attached prosthesis.
h) Base Ring: A ring with evenly-spaced holes which allows the radial position of each panel to be adjusted independently and the mounting insert to be securely attached (see items h and i in figure 1, figure 2c). The outward angling of this ring (11°) forces the panels to naturally fan outward to facilitate donning and doffing.

**FIGURE 4.**
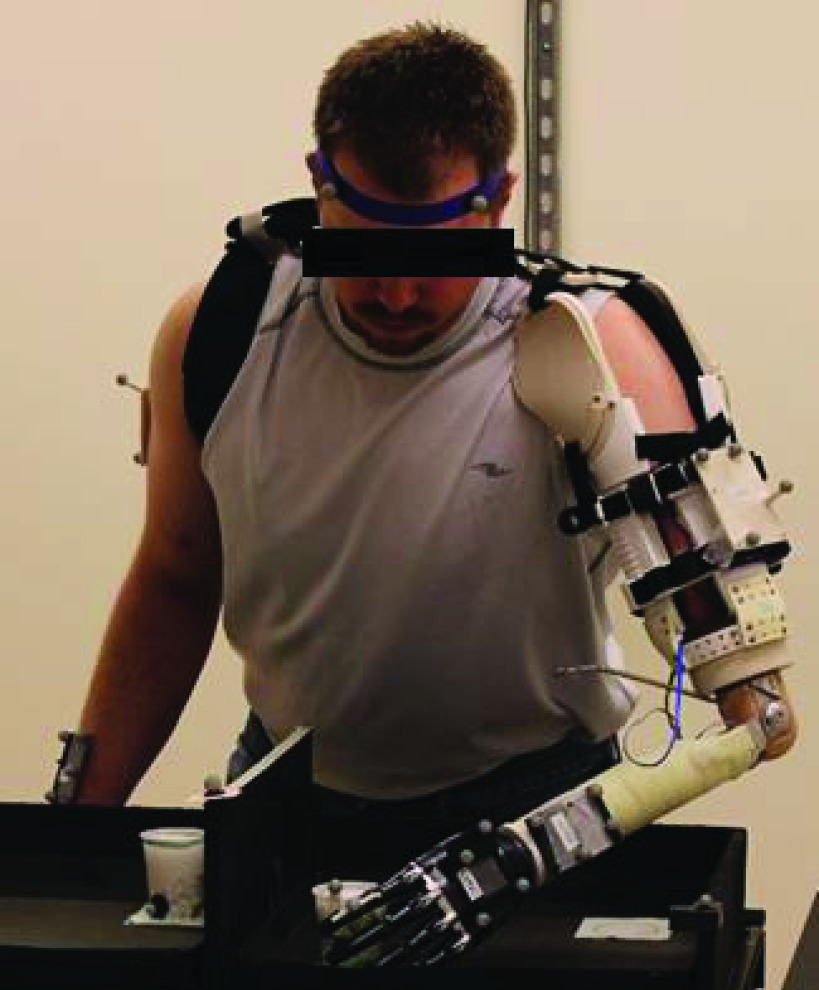
Participant performing Cup Transfer Task in a motion capture suite while wearing the MAPS system.

A Velcro® harness (figure 4) extends across the posterior shoulder and passes beneath the contralateral axilla. The harness meets with two Velcro® straps on the ipsilateral side, one attached to the proximal part of each shoulder panel, at a Y-shaped ring. This harness aids socket suspension and maintains contact between the panels and residuum. In addition to modular components that are interchangeable with others of varying sizes, the position of components can be adjusted to optimize suspension and user comfort. Figure 2a illustrates adjustment of the radial position and internal/external rotation of the shoulder panels, in addition to rotation about the anterior-posterior axis of the user.

### B. Participants

Participant 1, A 32-year-old male with a left transhumeral amputation performed 6 years prior volunteered to participate in the testing of the MAPS. The length of the participant’s residual limb was 24.3 cm and he wore a suction socket with BOA-clamped electrodes [25]. The participant’s prosthesis consisted of a mechanical elbow joint (ErgoArm plus, Otto Bock Inc., Germany) and a powered myoelectric hand (Bebionic Hand, Otto Bock Inc., Germany). Written informed consent was obtained from the participant and the protocol was approved by the University of Alberta Health Research Ethics Board (Pro00034663).

A duplicate thermoplastic suction socket was created based on the participant’s existing socket. Modifications were made by a prosthetist from the Glenrose Rehabilitation Hospital to achieve a comfortable fit for the user. A prosthesis was constructed to match, as closely as possible, the weight and dimensions of the participant’s usual socket-prosthesis system, including an identical elbow and hand system. Two-site proportional control of hand/open and close was achieved using two electrodes (13E200 MyoBock electrodes, Otto Bock Inc., Germany), one each placed on the biceps and triceps of the participant’s residual limb.

Participant 2, A 73-year-old participant with a right transhumeral amputation performed 14 years prior, who had also received Targeted Muscle Reinnervation was recruited to participate in preliminary assessment of myoelectric control. The length of the participant’s residual limb was 15.8 cm and his personal socket was a suction socket.

### C. Experimental Protocol

Assessment of the functionality of the sockets took place across 3 experimental sessions (3 hours each). The first session tested the participant using the thermoplastic suction socket and contained three assessment blocks: 1) range of motion (ROM), 2) mechanical, and 3) functional assessments. The same procedure was repeated in the second session with the MAPS. The final session was a characterization of the pressure and load distribution across the limb-socket interface of the MAPS system.

#### 1) Sessions 1 and 2 Experimental Protocol

##### a: Range of Motion Assessment

A modified version of the upper-body motion capture cluster marker set described in [26] was used, with individual markers added on the lateral and medial aspects of the mechanical elbow, and the participant’s anterior and posterior acromion, and socket 10 cm distal from the upper trim lines of the distal socket assembly, in accordance with the socket motion analysis configuration described in [7].

The maximum range of shoulder motion attainable by the participant was assessed using an 8-camera OptiTrack Flex 13 motion capture system with a sampling frequency of 120 Hz (NaturalPoint, Inc. Corvallis, OR, USA). The participant was asked to actively move to the limits of their shoulder ROM and the maximum angular deviation from the participant’s neutral position (arm relaxed in standing) was assessed for shoulder flexion, extension (in the sagittal plane), abduction (frontal plane), horizontal adduction at 90° flexion (coronal plane), and combined internal/external rotation with the participant’s arm at their side in resting position. Each motion was completed sequentially with the participant returning to a neutral resting position between each. The full ROM assessment was completed three times with the prosthesis fully extended, and three times with the elbow at 90 degrees in flexion.

##### b: Mechanical Assessment

Loads and moments in excess of those resulting from common assessments of upper-limb prosthesis control [27] were exerted on the donned socket system, and any resulting slip with respect to the skin was recorded. T-shaped markings on both the socket and participant’s residual limb allowed rotational and axial slipping to be measured. Axial forces were exerted upon the socket and gradually increased to a pre-defined maximum of 6 kg (59 N). Forces were applied and measured by exerting a downward force on a mechanical force gauge connected to the base plate of the socket via a 3D printed adapter. This adapter used the same threaded attachment system as the ErgoArm plus mechanical elbow. Moments were exerted by increasing the load exerted on a spring scale placed on a bar 0.5 m from the socket base plate, until the participant was unable to counter the moment. Each force or moment was exerted 3 times and held for 5 seconds, with slip recorded after each load cycle. Applied forces were measured.

##### c: Functional Assessment

To expose the participant to both gross motor movement and fine grasp control while using each socket, the participant completed 10 trials of the Cup Transfer Task [28]. The participant was asked to move 2 cups placed on a table over a barrier and across the participant’s midline, and then back to the starting position, resulting in 4 cup movements. The cups were compliant and filled with beads to simulate water, necessitating precise grip force modulation to avoid crushing and proper positioning to avoid spilling beads. Elbow flexion and wrist rotation were locked in positions chosen by the participant to perform the task. The task required the participant to reach varying distances from the body due to near and far placements of the cups, which produced a varied moment arm on the socket to challenge the effect of socket weight and stability on user control.

#### 2) Session 3 Experimental Protocol

Load distribution at the MAPS and residual limb interface was assessed using the TekScan VersaTek System and 9811E sensors (TekScan Inc., Boston MA). Following the protocol described in our previous work [8], two sensors were placed to span the posterior, lateral, and anterior regions of the residual limb and adhered with tape. A custom pressure-measuring bladder and pushing head identical to that used in [8], was used to equilibrate and calibrate the TekScan sensors while on the residual limb, as this has been shown to increase accuracy [29]. Three equilibrium points including a zero-pressure point were captured in addition to two calibration points which resulted in a saturation pressure of 8.78 and 9.0 kPa for the two sensors. 500 pressure readings across each sensor’s 92 sensels were obtained with a sampling rate of 50 Hz.

A pressure map of the participant’s residual limb was obtained by placing reflective motion capture markers on top of pressure sensors and interpolating from these points obtained from a static measurement in a motion capture suite. Interface pressure readings were taken in three poses: 1) without any prosthetic components attached, arm resting by the user’s side in neutral; 2) prosthesis attached with the elbow flexed and participant’s shoulder in neutral; 3) prosthesis attached with elbow extended and participant’s shoulder abducted to 90 degrees in-plane with the scapula. Readings in the last two positions were taken with and without a 1 kg mass held by the prosthetic end effector. Pressure maps were obtained by averaging the pressure readings and mapping these pressure regions to corresponding points on a mesh created from motion capture data using F-Socket clinical software (TekScan Inc., Boston MA).

#### 3) Preliminary Assessment of Multi-Site Myoelectric Control

To validate the MAPS system’s capacity to acquire EMG signals from multiple muscle sites, a preliminary assessment of myoelectric control was conducted. Participant 2, who had previously received a transhumeral amputation and Targeted Muscle Reinnervation, and who possessed experience with multi-site, multi-DOF control, was recruited solely to participate in this pilot study. Four muscle bodies on the biceps and triceps capable of voluntary contraction were identified and marked for electrode placement. The MAPS was configured with four electrodes: two in the interior panels and two connected to the circumferential straps. In the acquisition phase, the participant was asked to match four gestures presented on-screen: wrist pronation/supination, and wrist flexion/extension, in addition to rest. Each gesture was repeated three times with data acquired for 3 seconds. A linear discriminant analysis classifier was trained using time domain features (mean absolute value, zero crossing, wavelength, and slope sign change) and used as the predictor of intended gestures. In the testing phase, the participant was asked to match each of the four previously recorded gestures within a 15 second completion window, with 2 repetitions. Performance was assessed offline using a 3-fold cross-validation.

### D. Outcome Measures

The following quantitative and qualitative measures were analyzed:

1. Range of motion assessment: Total ROM (°) during active shoulder movement to determine the relative restrictions on shoulder motion imposed by the socket.
2. Mechanical assessment: Axial force (N) and torque ($\text{N}\cdot \text{m}$) to determine the maximum load that a socket worn by the participant may hold before slipping.
3. Functional assessment:
  3.1. Completion Rate (%) was defined as the percentage of the successful cup transfers.
  3.2. Mean Completion Time (s) was defined as the average time taken to successfully transfer all 4 cups.
  3.3. Relative Phase Duration (%) indicated the percentage of time spent on each phase of a movement, i.e., reach, grasp, transport and release.
  3.4. Shoulder Movement (°) consisted of total ROM of shoulder abduction/adduction, flexion/extension, and rotation during each movement of the Cup Transfer Task, and time-normalized joint angle trajectories with respect to initial starting limb orientation.
4. Pressure map (KPa) showed the distribution of pressure caused by the MAPS on the residual limb.
5. User survey collected at the beginning of the first session to assess participant’s own socket, and after the last assessment block for each of the suction and the MAPS.
  5.1 Orthotics and Prosthetics User’s Survey (OPUS) Satisfaction with device sub-score was used as an indicator for prosthesis fit and comfort [30]. High score indicates higher satisfaction with socket comfort and fit. Questions related to aesthetics and self-donning were excluded as they were not relevant to the intended use in myoelectric control evaluation.
  5.2 National Aeronautics and Space Administration Task Load Index (NASA-TLX) was used to assess user’s perceived physical and mental exertion when performing a task [31].

## III. Results

The main goal of this study was to develop a method to facilitate research in transhumeral prosthesis control and feedback through designing and objectively assessing a 3D-printed MAPS that is inexpensive to build and easy to use in a lab setting. We conducted various assessments to ensure that the developed socket enabled similar performance as a PSS.

### A. Range of Motion Assessment

**FIGURE 5.**
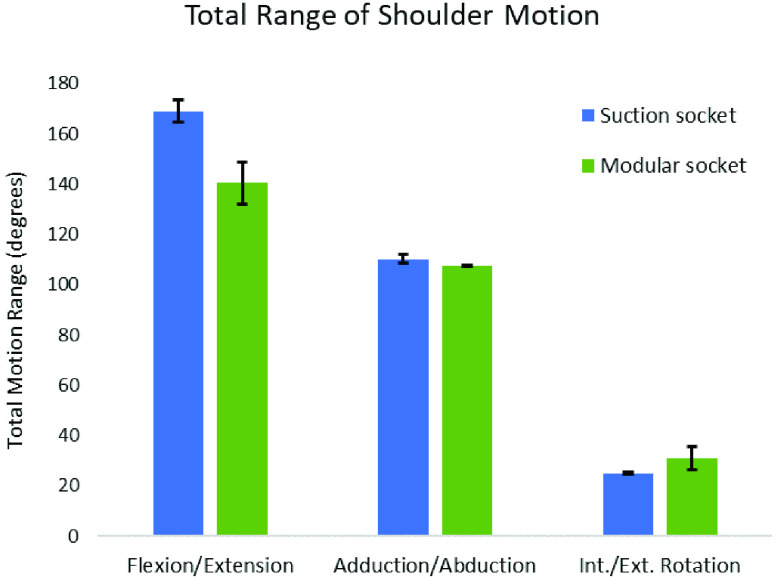
Total Range of Shoulder Motion for the MAPS and suction socket.

Assessment of active shoulder ROM revealed that the MAPS resulted in lower total range of shoulder flexion/extension (140.4 ± 8.23°) compared to the suction socket (166.5 ± 6.5°). Total range of abduction/adduction for the suction socket (109.6° ± 4.1) was similar to that of the MAPS (107.6 ± 0.25°), and the MAPS had a greater range of internal/external rotation (31.1 ± 4.5°) than the suction socket (24.8 ± 0.45°) (Figure 5). Both sockets enabled a total ROM exceeding that required for standard assessments of upper-limb myoelectric control [7], [32].

### B. Mechanical Assessment

Both sockets sustained repeated axial loading at 29.4 ± 1.0 N and 58.9 ± 1.0 N while worn by the participant, with no detectable slip. Comfortable axial torque limits with less than 1 mm of axial slip were similar for both sockets ($3.1~\pm ~0.5~\text{N}\cdot \text{m}$ and $2.6~\pm ~0.5~\text{N}\cdot \text{m}$ for MAPS and suction socket, respectively).

### C. Functional Assessment

The participant had a slightly higher completion rate for the Cup Transfer Task when using the suction socket (70%) than when using the MAPS (67%), and the completion time when using the suction socket was lower (43.6 s ± 5.1 s) than when using the MAPS (46.8 s ± 4.4 s). The average total trial durations of each socket fell within one standard deviation of the other.

**FIGURE 6.**
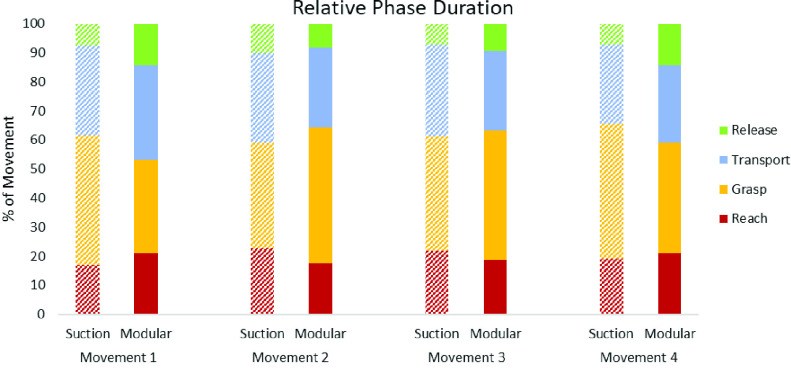
Average relative phase duration for each socket grouped by movement.

The average relative phase durations using the two socket types across all 4 cup transfer movements is shown in Figure 6. The differences in mean time-normalized reach, grasp, and transport times across all cup movements were similar for both sockets. The time-normalized duration of grasp phase when moving the cup closer to the body (movements 1 and 4) were shorter with the MAPS (32.1 ± 11.2% and 37.9 ± 11.0%) compared to the suction socket (44.4 ± 11.1% and 46.3 ± 12.3%). This trend was reversed for movement 2 and 3 of the cup placed further away from the body (46.9% ± 10.0% and 44.6 ± 10.5% for the MAPS versus 36.3 ± 10.3% and 39.3% ± 10.7%) for the suction socket).

**FIGURE 7.**
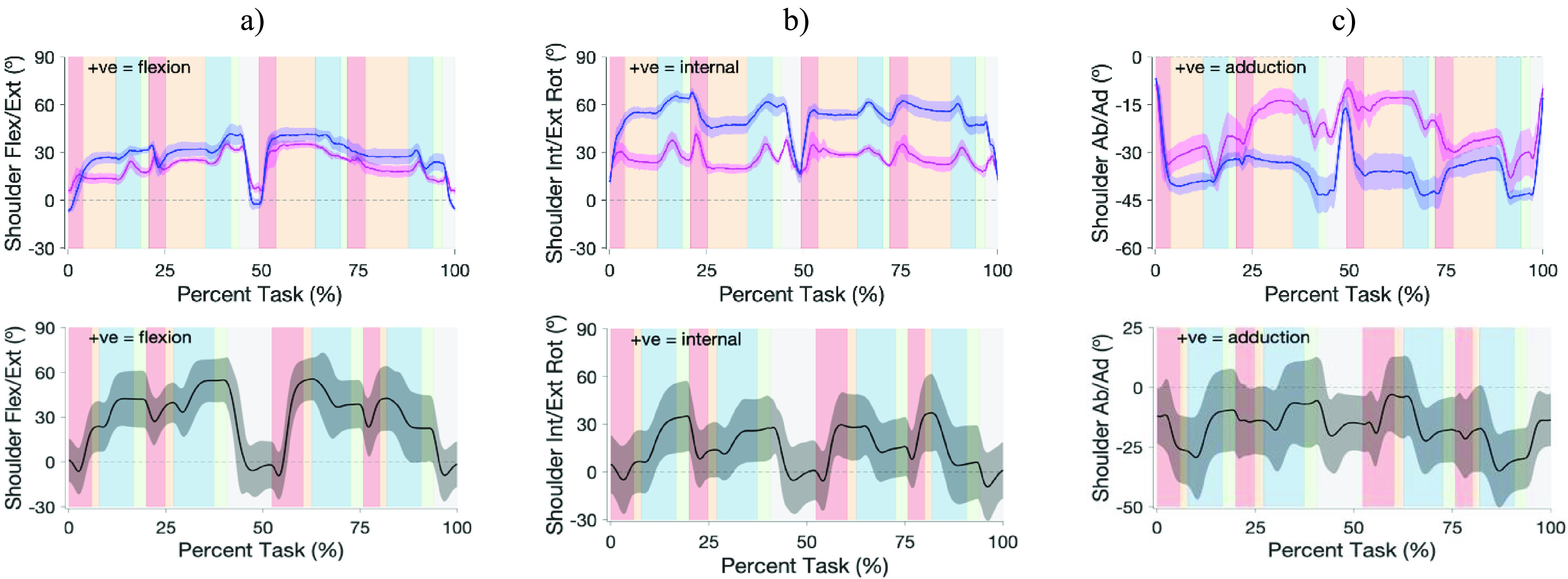
Top graphs: Shoulder a) flexion/extension, b) internal/external rotation, and c) abduction/adduction angle for the suction socket (pink) and the MAPS (dark blue), normalized to percent task completion for the cup transfer task. Lower graphs: equivalent normative shoulder angular kinematic graphs for the same task; black line represents normative mean and gray shade between-participant standard deviation. Red, orange, blue, and green vertical bars denote reach, grasp, transport, and release phases, respectively. Note that these phase lengths vary from prosthesis to normative data mainly due to prolonged grasp times, hence comparisons to expected normative movements can be made within phase.

Shoulder movement patterns are shown in Fig 7, with normative reference data for comparison [33]. Compared to normative movement patterns, both sockets demonstrated similar patterns of shoulder flexion/extension throughout the functional task. Greater total functional ROM for the MAPS system, closer to normative values, was reflected in the ROM values for 3 of the movements (*see supplementary table S1*). The MAPS system had internal/external rotation movements similar to normative movements pattern, whereas the suction socket seemed to have dampened rotation peaks. Abduction/ adduction movements were variable between sockets, with no consistent pattern from normative.

### D. User Survey

The OPUS survey results (Appendix B) indicated that all 3 sockets had comparable reported fit and comfort. The MAPS was reported to be more durable and more pain-free to wear than the other 2 sockets, with less abrasion and irritation compared to the suction socket. The participant’s own BOA socket was rated to have the most manageable weight, although all sockets were positively rated.

NASA-TLX results (Appendix C) indicated that use of either the MAPS or suction socket was reported as requiring similar physical and time demands and resulted in similar (low) levels of frustration. Using the MAPS was reported as being more mentally demanding, requiring more effort, and having lower performance compared to the suction socket.

### E. Maps- Residual Limb Pressure Map

**FIGURE 8.**
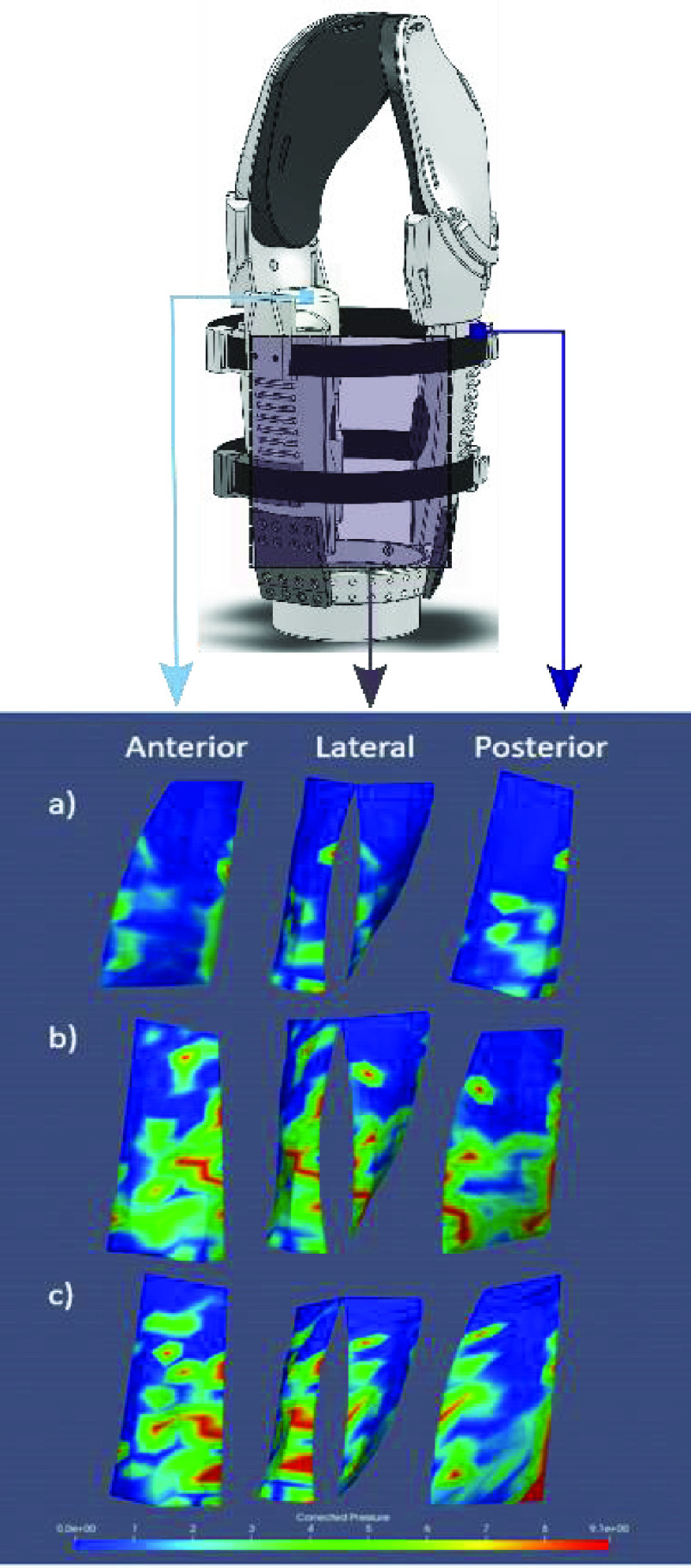
Pressure sensor configuration for (from left to right) front, lateral, and rear panel of the MAPS. Loading configurations: a) no prosthesis components attached, arm resting at side; b) prosthesis attached, elbow at 90 degrees flexion with 1kg load applied downward at end effector, and c) prosthesis attached and elbow extended with shoulder abducted to 90 degrees with 1kg load applied at end effector.

A map of the pressure distribution caused by the MAPS on the residual limb shows compression generally evenly distributed across each of the 3 panels in 3 limb positions (Figure 8.a). The magnitude of average pressure ranged from 2.19 kPa with no prosthesis attached to 3.53 kPa with 1 kg load at the prosthesis with elbow flexed to 90°. Pressure varied between the three panels depending on the orientation and loading condition. With increased loading in each of the loading positions, the compressive pressure increased across all panels. With no prosthetic components attached, average pressure at the limb-socket interface was 0.735 kPa in the anterior-lateral region and 1.069 KPa along the posterior region. Saturation of the sensors was reached at 8.78kPa. In the two most extreme loading conditions (shoulder abducted to 90 degrees in-plane with scapula and elbow flexed to 90 degrees, each with 1kg load), total area exceeding saturation value (>8.5kPa) was 4.70 cm^2^ and 2.28 cm^2^, respectively.

Two pressure concentrations were observed across the various loading configurations. The first was in the lateral region along the length of the residual limb when the arm was abducted 90 degrees and held in-plane with the scapula with a 1kg load attached to the end effector (Figure 8. B). Average pressure across the socket-residual limb interface in this position was 2.43 kPa. The second region of high pressure was observed with the elbow flexed to 90 degrees and a 1 kg load held by the end effector (see Figure 8. C). In this position, the load was distributed across the posterior-lateral region (2.67 kPa) with an increase in pressure near the posterior of the residual limb (3.33 kPa).

### F. Multi-Site Myoelectric Control Evaluation

**FIGURE 9.**
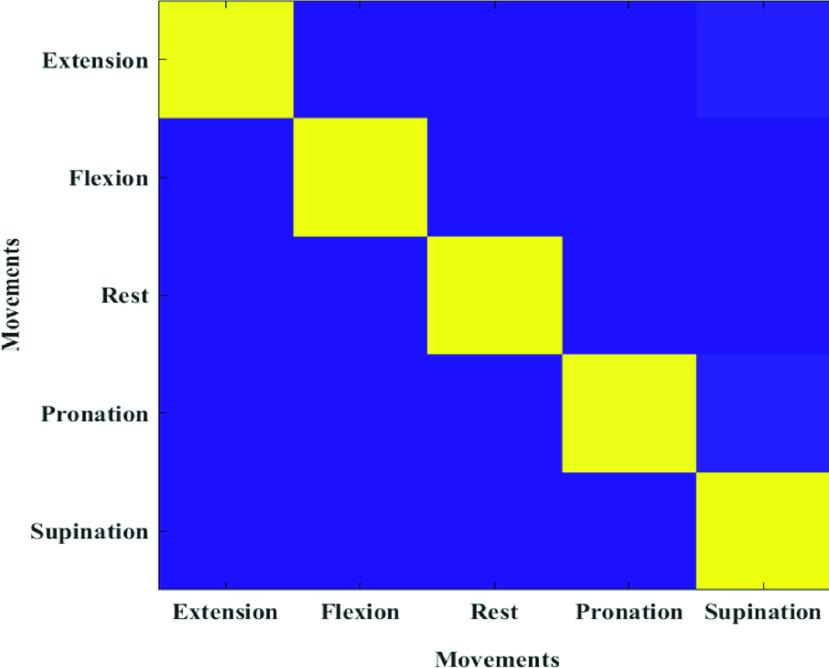
Confusion matrix for the LDA classifier trained on 5 classes.

Figure 9 shows the confusion matrix for participant 2 using the MAPS. It shows that 3 out of five classes were predicted with 100% accuracy and wrist extension was incorrectly predicted as supination in less than 10% of the movements.

## IV. Discussion

In this work, we developed a modular adaptable transhumeral socket with an adjustable interface that can be adapted to accommodate changes on the residual limb of a user, be used between different users, and accommodate typical myoelectric control strategies for in-lab testing. For the proposed MAPS system to be a viable alternative for use in experimental evaluation, differences attributable to the design of the socket must be identified so that results from future experiments utilizing the modular design can be reasonably generalized to the use of participant-specific sockets.

Both sockets tested in this work supported static loads in excess of what occurs in conventional control validation [27], [34]. Furthermore, the distribution of pressure at the MAPS-residual limb interface was qualitatively comparable to trends reported in existing literature [8], with normal but evenly distributed increases in pressure observed due to reaction forces countering the moment of the applied loads. Because the saturation value for both pressure sensors was lower than the 12.5kPa threshold used in [8], it is difficult to compare relative areas subjected to this level of pressure. However, despite the lower saturation threshold, the areas of saturation for the two most intense loading conditions (elbow flexed at 90 degrees with 1kg load and abducted in-plane of scapula with 1kg) of load fell within those reported in [8] for each respective loading condition. Therefore, the pressure distribution at the socket-residual limb interface is qualitatively and quantitatively similar to that reported with transhumeral PSS systems under similar loading conditions. When comparing to past work it should be noted that the areas reported to exceed saturation pressure threshold are only approximate, as the resolution of the residual limb mesh differs due to differing methods of point-cloud acquisition.

Functional differences in shoulder ROM were observed between use of the suction socket and the MAPS. Prosthesis users have been shown to generally have less shoulder movement compared with normative users when performing functional tasks [35]. Compared to the MAPS, maximum active shoulder ROM with the suction socket was greater for flexion/extension, similar for abduction, and less for internal/external rotation; however, during the functional task performance of the Cup Transfer Task, use of the MAPS involved a greater range of flexion/extension, and internal/ external rotation patterns more similar to normal movement. Abduction ranges and movement were similar between the two sockets. These findings suggest that both sockets enable a comparable functional range during task performance.

Reaching and transporting the cups required gross movement of the shoulder, and demonstrated comparable stability of the two socket systems in translating motion from the residuum to the positioning of the prosthesis, as indicated by the changes in all three shoulder movements within the red (reach) and blue (transport) phases (see figure 7). We also assessed slippage during the functional task and found that it was minimal (0.5–3 mm), which suggests that the attaching mechanism (panels and Velcro straps) does not allow the residual limb to move inside the socket except for the distal end of the residual limb, which is supported by the distal support when limb is relaxed but moves around when contracting. Tightening the circumferential Velcro straps may mitigate this issue but at the expense of comfort to the user.

In contrast, grasping an object requires consistent myoelectric control, dependent on adequate socket-skin electrode contact. Participants using the MAPS had shorter grasp times when working close to the body, and longer grasp times when grasping further from the body, in comparison to when using the suction socket. These differences were contrasted by the opposite trend in release times, thereby creating minimal differences in the participant’s overall performance in the aggregate of these phases. This indicates comparable myoelectric control performance between sockets under similar conditions but may also suggest a loss of electrode contact (and therefore less precise control) with the MAPS when reaching away from the body.

The user feedback surveys indicated that the MAPS comfort, fit, weight, and durability all scored comparably to that of the suction socket. Lower scores relating to skin abrasion were assigned to the participant’s own socket and the duplicate suction socket, which was attributed to pinch points near the axilla. Such pinch points were absent from the MAPS, which was reflected in both the pressure distribution map and user survey. This finding supports the correlation between pressure distribution and reported socket discomfort, as suggested in [36], in addition to the usefulness of pressure maps in predicting socket comfort.

Surprisingly, performance as perceived by the participant was lower with the MAPS compared to the suction socket and was reported to require higher mental effort despite similar completion times and completion rates between the two sockets. The discrepancy between objective and perceived measures of performance reflects previous findings where changes in the participant’s understanding of a task due to external factors are not represented by objective performance outcomes [37]. In the context of this experiment, the factor likely attributable to this change in task perception is the participants’ unfamiliarity with the new system, compounded with a delay between the first and second testing sessions. In fact, the participant had gone for several months without his myoelectric prosthesis (which was under repair) between the first and second sessions and may therefore have had lower confidence overall with his performance. Future studies using the MAPS system planning to elicit similar measures of participant perception should take into consideration possible impacts of home prosthesis use prior to laboratory testing and include similar acclimatization times for new systems.

As only one participant was recruited for functional assessments in this study, the results cannot be generalized as the effects of factors including residual limb shape, size, and length on socket fit and performance have yet to be examined. This MAPS system was configured for users with a relatively long residual limb (24.3 cm for P1 and 15.8 cm for P2); the efficacy of using different part sizes in creating a well-fitting and functional socket for the majority of the transhumeral population must still be assessed. Evaluation of prosthesis control should also be extended to tasks involving 2- and 3-DOF movement. A larger sample size would provide a more complete assessment of the socket’s ability to accommodate multiple electrodes from which signals to predict different intended actions can be clearly discerned.

Future work should leverage the socket’s modular design and stable platform to integrate sensory feedback systems which interface directly with a user’s residual limb, thereby enabling assessment of closed-loop prosthetic control. A similar system could also be developed for transradial amputation. Extension to lower-limb amputations would require structural reinforcement and, likely, stronger materials to support the weight of a human adult without breaking, as well as accommodation of higher contract forces.

## V. Conclusion

A 3D-printed modular, adaptable prosthetic socket was developed and assessed against a conventional suction socket in a case study assessing differences in mechanical or functional performance and user experience. Both sockets met static loading requirements and yielded similar functional outcomes. While there was variation between the sockets in terms of overall performance metrics such as completion time and some kinematic features, no clear trends emerged favouring one socket over the other. User feedback was similar for both comfort and exertion between the two sockets. Overall, this modular adaptable socket system demonstrates potential for replacing participant-specific sockets in future control strategy evaluation experiments.

## Conflict of Interest

The authors declare no conflict of interest.
